# Differential Immune Infiltration Profiles in Colitis-Associated Colorectal Cancer versus Sporadic Colorectal Cancer

**DOI:** 10.3390/cancers15194743

**Published:** 2023-09-27

**Authors:** Josefine Schardey, Can Lu, Jens Neumann, Ulrich Wirth, Qiang Li, Tianxiao Jiang, Petra Zimmermann, Joachim Andrassy, Alexandr V. Bazhin, Jens Werner, Florian Kühn

**Affiliations:** 1 Department of General, Visceral and Transplant Surgery, Ludwig-Maximilians-University Hospital Munich, 81377 Munich, Germany; 2 Department of Colorectal Surgery and Oncology (Key Laboratory of Cancer Prevention and Intervention, China National Ministry of Education & Key Laboratory of Molecular Biology in Medical Sciences), The Second Affiliated Hospital, Zhejiang University School of Medicine, Hangzhou 310058, China; 3 Zhejiang Provincial Clinical Research Center for CANCER & Cancer Center of Zhejiang University, Zhejiang University School of Medicine, Hangzhou 310058, China; 4 Department of Pathology, Ludwig-Maximilians University, 81377 Munich, Germany; 5 German Cancer Consortium (DKTK), Partner Site Munich, 80336 Munich, Germany

**Keywords:** Crohn’s disease, colitis-associated colorectal cancer, ulcerative colitis, inflammatory bowel disease, immune infiltrate, T-cell exhaustion

## Abstract

**Simple Summary:**

Chronic inflammation plays a significant role in colorectal cancer (CRC) development, particularly in colitis-associated CRC (CAC). This study examined immune infiltration patterns in CAC patients compared to sporadic CRC (sCRC) patients and their impact on prognosis. Twenty CAC and twenty sCRC patients, matched by tumor characteristics, were analyzed. Immunohistochemistry targeted various immune markers, including T-cell and B-cell markers, in tumor and adjacent mucosal tissues. The results revealed differences between CAC and sCRC in T-cell exhaustion markers (TOX and TIGIT) and immune cell infiltration. High CD3+ and CD20+ cell levels correlated with improved survival in CAC but not in sCRC. This study highlighted distinct immune profiles in CAC and sCRC, suggesting that T-cell exhaustion might play a different role in CAC development than in sCRC. Understanding these immune differences could impact treatment strategies and prognosis for CAC patients.

**Abstract:**

Background: Chronic inflammation is a significant factor in colorectal cancer (CRC) development, especially in colitis-associated CRC (CAC). T-cell exhaustion is known to influence inflammatory bowel disease (IBD) progression and antitumor immunity in IBD patients. This study aimed to identify unique immune infiltration characteristics in CAC patients. Methods: We studied 20 CAC and 20 sporadic CRC (sCRC) patients, who were matched by tumor stage, grade, and location. Immunohistochemical staining targeted various T-cell markers (CD3, CD4, CD8, and FOXP3), T-cell exhaustion markers (TOX and TIGIT), a B-cell marker (CD20), and a neutrophil marker (CD66b) in tumor and tumor-free mucosa from both groups. The quantification of the tumor immune stroma algorithm assessed immune-infiltrating cells. Results: CAC patients had significantly lower TOX+ cell infiltration than sCRC in tumors (*p* = 0.02) and paracancerous tissues (*p* < 0.01). Right-sided CAC showed increased infiltration of TOX+ cells (*p* = 0.01), FOXP3+ regulatory T-cells (*p* < 0.01), and CD20+ B-cells (*p* < 0.01) compared to left-sided CAC. In sCRC, higher tumor stages (III and IV) had significantly lower TIGIT+ infiltrate than stages I and II. In CAC, high CD3+ (*p* < 0.01) and CD20+ (*p* < 0.01) infiltrates correlated with improved overall survival. In sCRC, better survival was associated with decreased TIGIT+ cells (*p* < 0.038) and reduced CD8+ infiltrates (*p* = 0.02). Conclusion: In CAC, high CD3+ and CD20+ infiltrates relate to improved survival, while this association is absent in sCRC. The study revealed marked differences in TIGIT and TOX expression, emphasizing distinctions between CAC and sCRC. T-cell exhaustion appears to have a different role in CAC development.

## 1. Introduction

Crohn’s disease (CD) and ulcerative colitis (UC) are the most common entities of inflammatory bowel disease (IBD). They are associated with high morbidity and, in severe cases, increased mortality [1]. IBD patients have an increased risk of developing colorectal cancer (CRC) compared to the normal population [2]. IBD patients who have colitis-associated colorectal cancer (CAC) have poorer overall survival (OS) compared to non-IBD patients; in the comparison between UC-associated CRC patients and CD-associated CRC patients, it was observed that UC-associated CRC patients exhibited superior overall survival (OS) outcomes. Additionally, CAC was distinguished by aggressive tumor characteristics, including a higher prevalence of right-sided tumors [3].

Chronic inflammation promotes the development of CRC and is considered the major driver of CAC [4], but the mechanisms involved remain unclear [5]. The critical role of immune function in the tumor microenvironment is recognized [6]. Immune profiling contributes to prognostic assessment in terms of disease-free survival (DFS) and OS and evaluates treatment efficacy by identifying potential targets of immunotherapies [7].

CAC follows a distinct trajectory characterized by the evolution from low-grade dysplasia to high-grade dysplasia. This progressive transformation occurs within the context of chronic inflammation and presents unique challenges for diagnosis and treatment [5]. In contrast, sCRC originates from adenomatous polyps, gradually advancing from benign adenoma to invasive cancer. This stepwise progression necessitates thorough surveillance and early intervention to mitigate the potential for malignancy [8]. Immunophenotyping is widely established for sporadic (s)CRC, whereas CAC has only been poorly typed. Moreover, there has been increasing evidence that T-cell exhaustion in the mucosa of IBD patients is related to inflammatory disease progression [9,10,11]. T-cell exhaustion is characterized by loss of effector function, including proliferation, release of cytokines, and secretion of cytolytic molecules [12]. McKinney et al. investigated the correlation between CD8+ T-cell exhaustion and IBD and found it to be associated with a favorable prognosis in the context of IBD inflammatory activity [9]. Corridoni et al. discovered cell clusters in UC mucosa with exhausted CD8+ T-cell characteristics, including co-inhibitor molecule and transcription factor expression [11,13]. Similarities between TILs in CRC and the immune profiles of colonic T-cells in IBD were recently demonstrated [11]. The correlation between exhausted CD8+ T-cells in the colonic mucosa of IBD patients and their counterparts within the tumor microenvironment, particularly in CAC progression, still needs to be more adequately understood [11].

There is an extensive body of research on immune cell infiltrates in IBD which are also increasingly influencing pharmacological treatment strategies. To this day, there are only a handful of studies that investigate the tumor immune infiltrate in CAC compared to sporadic CRC. The most comprehensive study on this topic was conducted by Soh et al. in 2019 [14]. This study revealed that the CAC group displayed significantly lower levels of CD3-, CD8-, Foxp3-, and PD-L1-expressing cells (all *p* < 0.01) compared to sCRC [14]. Notably, a high expression of CD3+ and CD8+ immune cells in CAC patients correlated with enhanced overall survival [14]. These findings underscore the marked differences in immune profiles between CAC and sporadic CRC patients, highlighting distinct disease characteristics. Consequently, immunoprofiling emerges as a promising tool for evaluating clinical prognosis in CAC.

Using different lymphocyte markers and CD66b for neutrophils in this study, we aimed to assess various aspects of immune infiltration and activation within the tumor microenvironment and address novel markers for T-cell exhaustion. These markers provide information about different immune cell populations and their functional status, such as T-cells (CD3, CD4, and CD8), regulatory T-cells (FOXP3), T-cell exhaustion (TIGIT, TOX), B-cells (CD20), and neutrophils (CD66b). In this project, in the context of CAC in UC and Crohn’s colitis, the T-cell populations presented here will be further investigated in tumor tissue in inflamed and non-inflamed areas of the colon. To minimize confounders, we conducted the first study on CAC and sCRC, precisely matching tumor location, TNM stages, and grading. By evaluating the expression and distribution of these markers, the study aimed to gain insights into the immune response and its potential impact on the development and progression of CAC and sCRC.

## 2. Materials and Methods

### 2.1. Population

This study enrolled CAC and sCRC patients who underwent surgical resection at the Ludwig-Maximilians-University Munich (LMU) Hospital (Munich, Germany) between November 2010 and 2019. Patients with CAC were diagnosed with colorectal carcinoma in the resected specimens of underlying IBD. The median duration from the diagnosis of IBD to CAC was 18 years (0–39 years). To exclude the potential confounding factors associated with the tumor immune infiltrations, we matched 20 CAC patients with 20 sCRC patients according to their TNM status, tumor grade, and tumor location (right- vs. left-sided). SCRCs were identified from the CRC cohort by excluding hereditary CRC and CAC. The exclusion criterion was a known familial predisposition, such as variants associated with non-polyposis colorectal cancer (HNPCC); polyposis variants, e.g., Familial Adenomatous Polyposis (FAP); or MUTYH-associated syndromes. The diagnosis of recruited patients was histologically confirmed after resection. Baseline demographics and clinical data (sidedness of CRC, histopathological classification of the resected specimen, the residual tumor classification of resection, comorbidities, hospital days, survival, and clinical test parameters) were retrospectively retrieved from medical records. This study was approved by the LMU Munich ethics committee (No. 21-0323) and was conducted in accordance with the Declaration of Helsinki.

### 2.2. Immunohistochemistry

Three-micrometer sections of paraffin-embedded tissues from enrolled patients were provided by the pathology department and our clinic’s biobank (Biobank i.A. HTCR). A pathologist first screened the corresponding tissues to confirm the entity of tumor slides and inflammatory state. Inflamed tissue sections were available in *n* = 13 cases.

Immunohistochemical staining for CD3, CD4, CD8, FOXP3 (forkhead box P3), TIGIT (T-cell immunoreceptor with immunoglobulin and ITIM domain), TOX, CD20, and CD66b was conducted as previously described [15,16]. The slides were deparaffinized in xylene and rehydrated in successive incubations with decreasing ethanol concentrations (100%, 96%, 70%, and 0%). Then, we retrieved the antigens with 0.01 M citrate buffer (pH 6.0 for markers of CD3, TIGIT, TOX, CD20, and CD66b) or 0.001 M ethylenediaminetetraacetic acid (EDTA) solution (pH 8.0 for markers of CD8, CD4, and Foxp3), using 96 °C water baths, followed by the blocking of avidin and biotin (Vector Laboratories, Newark, CA, USA), and endogenous alkaline phosphatase (Vector Laboratories, Newark, CA, USA). Incubation of the slides with the primary antibody (see Supplementary Table S1) was conducted overnight at 4 °C or 1.5 h at room temperature (only for CD3). Following the 30 min incubation with the biotinylated secondary antibody (dilutions: 1:200 and 1:100 for TIGIT), the slides were stained with an ABC-AP kit (Vector Laboratories, Newark, CA, USA) and alkaline phosphatase substrate (Vector Laboratories, Newark, CA, USA). Nuclei were counterstained with hemalum solution (Sigma-Aldrich, St. Louis, MO, USA). Positive tissue controls and appropriate isotype control antibodies for each marker were used to confirm the specificity of primary antibodies.

The primary antibodies used in this study are listed as follows: anti-CD3, anti-CD4, anti-CD8, anti-FOXP3, anti-TIGIT, anti-CD20 (all Abcam, Cambridge, UK), anti-TOX (Sigma-Aldrich, St. Louis, MO, USA), and anti-CD66b (BioLegend, San Diego, CA, USA). Three isotype control antibodies were applied: IgG (Abcam, Cambridge, UK) for CD3, CD4, CD20, TIGIT, and TOX; IgM, κ (BioLegend, San Diego, CA, USA) for CD66b; and IgG1, κ (Abcam, Cambridge, UK) for CD8 and Foxp3. Biotinylated secondary antibodies, anti-mouse IgG antibody (BA-200), and anti-rabbit IgG antibody (BA-1100) were purchased from Vector Laboratories (Newark, CA, USA).

### 2.3. Quantitative Analysis of Immunohistochemical Staining

An algorithm for quantifying the tumor immune stroma (QTiS) was used to measure the level of immune-cell infiltrations on the slides of immunohistochemical staining [17]. The hotspot of staining markers was defined as the areas with the highest density of CD3+, CD4+, CD8+, Foxp3+, TIGIT+, TOX+, CD20+, and CD66b+ cells in a 200-fold magnification field. Firstly, three hotspots per marker on each slide were identified and captured using the BX40 microscope (Olympus, Tokyo, Japan) and AxioCam MRc5 digital camera (Zeiss, Oberkochen, Germany). Secondly, quantifying positive cells per image was performed using the color deconvolution of Fiji-ImageJ software (Version 1.53s, National Institutes of Health, Bethesda, MD, USA) with threshold adjusting. The mean value of three hotspots for each marker was considered to be the immune infiltration density on one slide.

### 2.4. Statistical Analysis

The statistical difference between the continuous variables was calculated using the independent *t*-test, Wilcoxon rank-sum test, or Kruskal–Wallis test, depending on a normal distribution. The Friedmann test was performed to assess the differences among continuous variables. The categorical variables were compared by applying Fisher’s exact test. *Survminer* R package (version 0.4.9) was used to select the best cutoff point for classifying the patient cohort into high- or low-infiltration groups for survival analysis (https://www.R-project.org/ (accessed on 23 December 2022)) [18]. The log-rank test was used to evaluate the survival difference between the two groups. Receiver operating characteristic (ROC) curves were made to assess the specificity and sensitivity of each marker on the overall survival of CAC and sCRC patients, respectively. The *p*-values < 0.10 were defined for inclusion of covariates in the multivariate Cox regression after the univariate Cox regression analysis. Overall survival refers to the time from the surgical resection to death. Statistics were performed using R (version 4.1.0), IBM SPSS Statistics version 26 (IBM, Armonk, New York, NY, USA), and Graph Pad Prism version 7 (GraphPad Software, Inc., New York, NY, USA). In general, *p*-values < 0.05 were considered statistically significant.

## 3. Results

### 3.1. Study Population and Patients’ Characteristics

The demographic and clinical characteristics of the study population are presented in Table 1. As expected, the CAC population was significantly younger [5] than the CRC population, with a mean age of 50.85 ± 15.48 years compared to 68.20 ± 11.52 years (*p* < 0.001). Other parameters, including localization, TNM stage, UICC stage, Charlson Comorbidity Index (CCI), and the lymph node ratio (proportion of affected lymph nodes out of all resected lymph nodes) did not exhibit significant differences between the CAC and sCRC group. Furthermore, the overall survival did not significantly differ between the groups (Supplementary Figure S1A).

### 3.2. Comparison of Total Cell Count between Normal-Appearing Mucosa and Tumor-Infiltrating Cells

**Figure 1:**
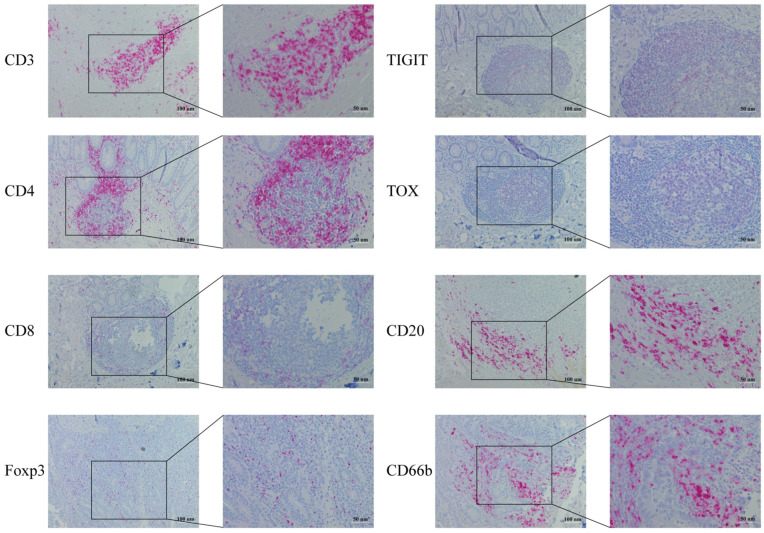
Representative immunohistochemistry images of eight tissue-infiltrated immune markers. The 100× magnification corresponds to the scale bar of 100 nm, while the 200× magnification corresponds to the scale bar of 50 nm.

**Figure 2:**
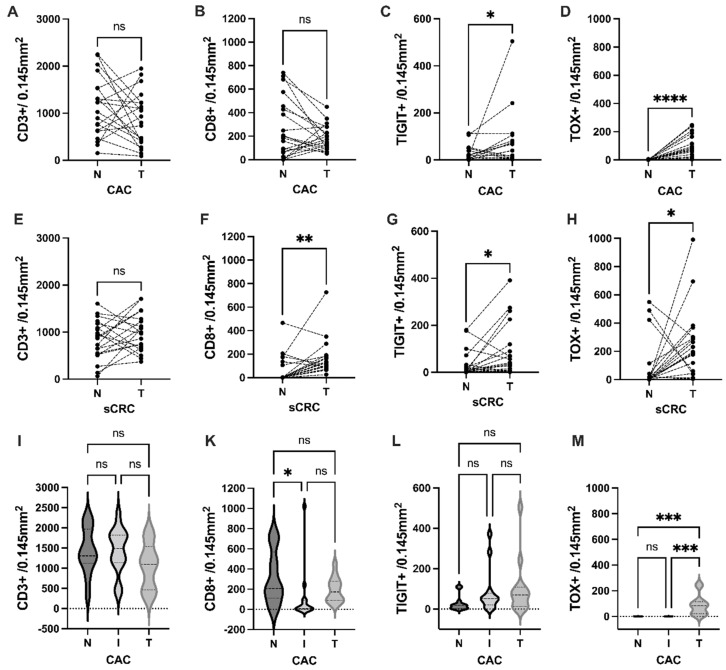
Comparison of CD3+ and CD8+ T-cell immune cell infiltrate between the tumor (T) and tumor-free “normal” mucosa (N) of *n* = 20 CAC patients (**A**–**D**) and *n* = 20 sCRC patients (**E**–**H**) Wilcoxon test). In *n* = 13 cases, inflamed tissue (**I**) was available for CAC, we compared the infiltrate between normal (N), inflamed tissue (I) and Tumor (T) (**I**–**M**) Friedmann test and Dunn’s multiple comparisons test were used with: ns, *p* ≥ 0.05; * *p* < 0.05; ** *p* < 0.01; *** *p* < 0.001; **** *p* < 0.0001).

The representative images of immunohistochemistry staining for eight immune markers are illustrated in Figure 1. An analysis of immune cell infiltration between tumor-free mucosa and tumor tissue in CAC and sCRC was conducted. CAC revealed no significant difference in the cell counts of CD3 and CD8 infiltrates (as demonstrated in Figure 2A and Figure 2B, respectively). However, it was found that CAC exhibited a significantly enriched presence of TIGIT (*p* = 0.011, as depicted in Figure 2C) and TOX (*p* < 0.0001, as shown in Figure 2D) in comparison to the corresponding tumor-free mucosa. In contrast, while the intratumoral count of CD3+ cells in sCRC were not significantly elevated (Figure 2E), there was a notable increase in the number of CD8+ infiltrates (*p* = 0.003, as depicted in Figure 2F). Additionally, there was a significant enrichment of TIGIT and TOX counts in sCRC (*p* = 0.036 and *p* = 0.041, respectively; Figure 2G,H).

For *n* = 13 patients with CAC, inflamed nontumoral tissue was also available. Upon comparing the corresponding tissues, a significant enrichment of CD8 infiltrate was observed in the normal mucosa (Figure 2K). However, no significant difference was identified in terms of CD3 and TIGIT (Figure 2I,L). In contrast, TOX was selectively enriched in the tumor tissue but not in the normal or inflamed tissue sections, as illustrated in Figure 2M.

### 3.3. Comparison of an Immune Infiltrate of Tumor Tissue and Normal Mucosa between CAC and sCRC

**Figure 3:**
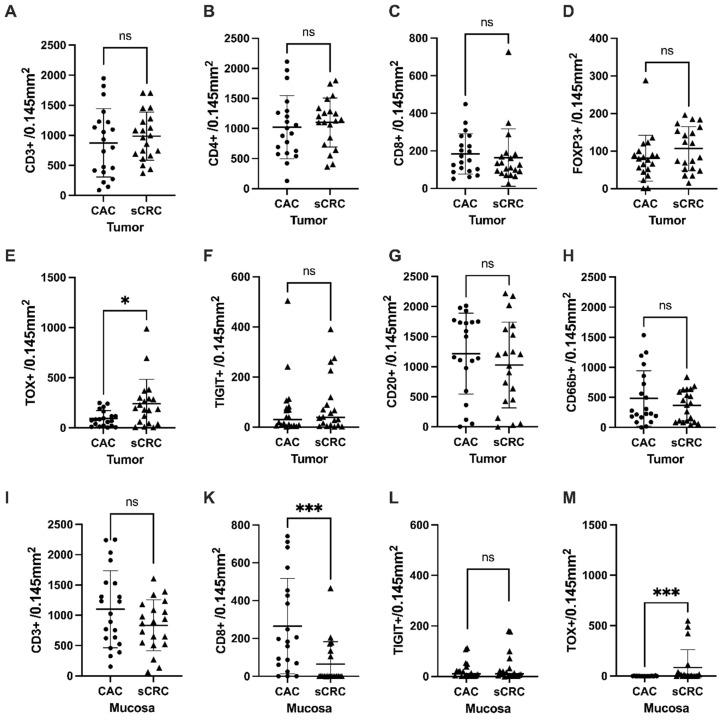
(**A**–**H**) Cell counts of tumor-infiltrating cells were compared between colitis-associated colorectal cancer (CAC) and sporadic colorectal cancer (sCRC) in *n* = 20 cases, (mean ± SD). (**I–M**) Cell counts of mucosa infiltrating cells were compared between CAC and sCRC. Mann–Whitney test was applied in all cases (ns, *p* ≥ 0.05; *, *p* < 0.05; ***, *p* < 0.001).

No significant differences were found when analyzing various intratumoral immune cells between CAC and sCRC, including all T-cells (CD3+), CD4+ and CD8+ T-cells, CD20+ B-cells, and CD66+ neutrophils (Figure 3A–D,G,H). Additionally, the distribution of TIGIT-positive cells did not exhibit any differential patterns between the two groups (Figure 3F). The only exception was the significantly higher abundance of TOX-positive cells in sCRC compared to CAC (Figure 3E).

Next, we examined the distribution between CD and UC; again, no significant difference was found regarding the distribution of the markers studied in TILs (Supplementary Figure S2A). A further analysis of the mucosa showed a substantial increase in CD8+T-cell infiltration in both CD and UC compared to sCRC (*p* = 0.028 and *p* = 0.014; Figure 3K and Supplementary S2B), however, no significant difference was found with regard to only CD3+ infiltrate and TIGIT+ cells (Figure 3I,L). Additionally, sCRC patients exhibited significantly more TOX-positive cells in the mucosa than UC and CD patients (*p* = 0.012 and *p* = 0.016; Figure 3M and Supplementary Figure S2C). The CD3+ infiltrate strongly correlated with the CD4+ infiltrate (Pearson *r* = 0.89 for CAC and *r* = 0.83 for sCRC; Figure S1B) but not with the CD8+ infiltrate.

#### The Distribution of Tumor-Infiltrating Cells concerning UICC Stage and Tumor Localization

**Figure 4:**
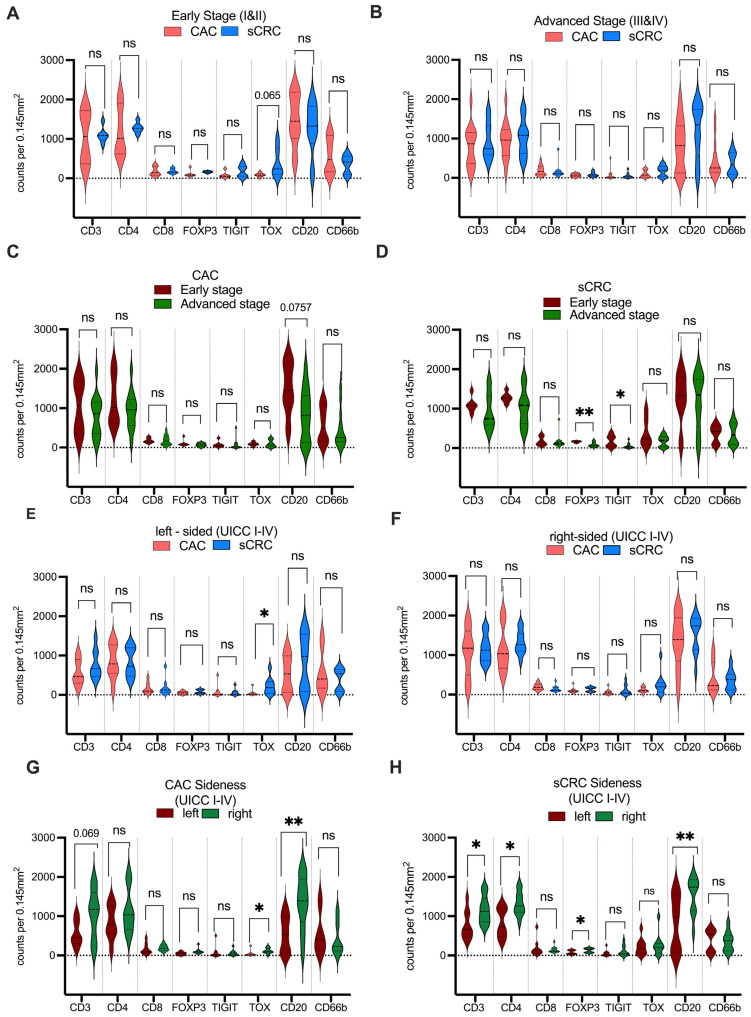
Violin plots demonstrating the immunohistochemical markers in sCRC and CAC patients. The cell counts of the early tumor stage between CAC and sCRC were compared (**A**), as well as those in the advanced tumor stage (**B**). Additionally, early and advanced cancer cell counts were compared in CAC patients (**C**) and sCRC patients (**D**). The analysis further compared left-sided tumors between CAC and sCRC (**E**) and those in right-sided tumors (**F**). The distribution of immune cells between right- and left-sided CAC (**G**) and sCRC (**H**) was also assessed. To determine the significance of the markers, the Mann–Whitney test was employed to compare the markers between CAC and sCRC (ns, *p* ≥ 0.05; * *p* < 0.05; ** *p* < 0.01).

The distribution of immune cells within TILs was found to be independent of the UICC stage based on the comparison of early-stage (UICC I and II; Figure 4A) and advanced-stage (UICC III and IV; Figure 4B) carcinomas, with no significant differences in cell counts observed between CAC and CRC. However, a nonsignificant trend towards an increased intratumoral TOX count was noted in early-stage sCRC (*p* = 0.065, as depicted in Figure 4D).

A further analysis of immune counts in CAC between early and advanced tumor stages showed a nonsignificant trend towards an elevation of B-cells in early-stage tumors (Figure 4C). In contrast, patients with sCRC exhibited elevated levels of FOXP3 in early-stage tumors (*p* = 0.009), as well as high levels of TIGIT (*p* = 0.026).

As commonly practiced, the right-sided and left-sided CRCs were divided up to the level of the splenic flexure of the colon [19].

Notably, left-sided sCRC has significantly higher levels of TOX+ cell infiltration than the counterparts of CAC (*p* = 0.03; Figure 4E). No differences were found in the immune infiltration between right-sided sCRC and CAC (Figure 4F). Patients with right-sided CACs exhibited a markedly higher count of TOX+ cells than those with left-sided ones (*p* = 0.0124). In addition, right-sided CAC patients had a significantly higher count of CD20+ B-cells than left-sided ones (*p* = 0.0096; Figure 4G). Furthermore, right-sided sCRCs exhibited significantly higher lymphocytes infiltration than left-sided ones (Figure 4H), including total T-cells (*p* = 0.020), B-cells (*p* = 0.006), CD4+ T-cells (*p* = 0.020), and FOXP3+ T-cells (*p* = 0.025).

### 3.4. Prognostic Analysis and Risk Factor Identification

A prognostic analysis was conducted on the eight markers. In the “best cutoff” analysis, it was found that CAC patients with a high CD3+ infiltration of TIL displayed a superior prognosis compared to those with high CD3+ infiltration. Furthermore, a high CD20+ infiltrate was associated with improved survival (*p* = 0.005). The computed “best cutoff values” for the immune scoring are depicted in Supplementary Table S2.

**Figure 5:**
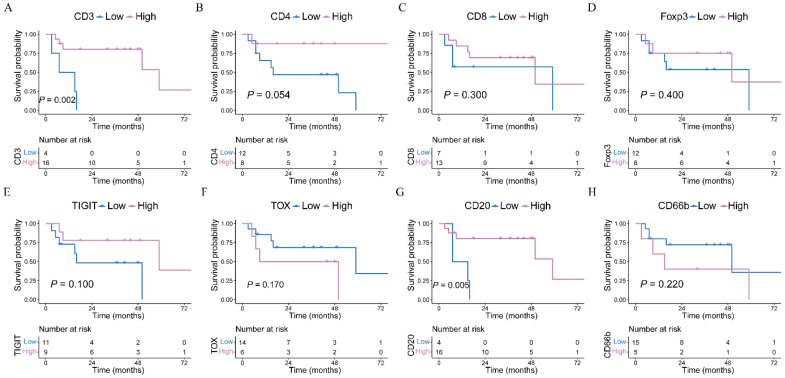
A prognostic analysis of immune infiltrations in colitis-associated colorectal cancer (CAC) patients after using the *Survminer R* package to select the best cutoff point for classifying the patient cohort into high- or low-infiltration groups. (**A**–**D**) Overall survival comparison between high-infiltration and low-infiltration of CD3+, CD4+, CD8+, and Foxp3+ immune cells in CAC patients, respectively. (**E**–**H**) Overall survival comparison between high-infiltration and low-infiltration of TIGIT+, TOX+, CD20+, and CD66b+ immune cells in CAC patients, respectively.

Regarding high CD4+ infiltrates, a nonsignificant trend was observed (*p* = 0.054) (Figure 5). However, the ROC analysis for assessing the predictive performance pertaining to the overall survival of patients did not yield a marker with adequate diagnostic power for predicting 1- or 3-year survival in CAC patients (Supplementary Figure S3).

In the case of sCRC patients, low CD8+ cell counts and low TIGIT+ cell counts were correlated with improved survival (log-rank test: *p* = 0.024 and *p* = 0.038, respectively). However, low CD4+ cell counts and high Foxp3+ counts only demonstrated a nonsignificant trend (see Supplementary Figure S4).

In the ROC analysis, the high CD3+, CD4+, and CD8+ counts and TIGIT+ and TOX+ counts showed sufficient AUC for predicting 1-year survival rates but not for 3-year overall survival rates (see Supplementary Figure S5).

In a univariate Cox regression analysis, CD3+ and CD4+ TILs and the lymph node ratio were identified as significant risk factors for mortality in CRC patients. Because tumor stage [20] and the Charlson Comorbidity Index (CCI) [21] are established risk factors in this population, we decided to include them in the multivariate analysis. However, our results did not confirm any of the abovementioned risks as independently associated with mortality (as seen in Table 2). In the case of sCRC, our analysis revealed that CD3+ levels and tumor stage were independent risk factors for mortality.

## 4. Discussion

The tumor microenvironment is characterized by numerous immunosuppressive cells and cytokines that may contribute to the development of exhausted CD8+ T-cells [22,23,24]. In the present study, we performed a comprehensive comparison involving 20 CAC patients who were matched with 20 sCRC patients based on their tumor characteristics. Our investigation encompassed the assessment of eight distinct immunohistochemical markers to elucidate potential variations between these two patient groups. Since the immune cell infiltrates in CAC patients may also be influenced by other factors, such as inflammatory activity [25], we also investigated infiltration in inflamed tumor-free mucosa in CAC patients.

Previous studies on the immune cell involvement in CRC have indicated that T-cell markers are linked to clinical outcomes [26]. We could not identify differences in the T-lymphocyte distribution between these groups, but CD3+ and CD4+ infiltrates were enriched in sCRC. Our data also showed that high levels of CD8+ cells in sCRC were related to worse survival, and high CD3+ cell counts could be identified as an independent risk factor. In contrast, for CAC, increased CD3+ cell counts were associated with significantly improved OS in the present study, but the multivariate analysis failed to confirm this as an independent risk factor.

Michael-Robinson et al. reported increased intratumoral CD8+/CD3+ counts, comparing 20 CAC to 40 sCRC samples, but with reduced Granzyme B expression and tumor cell apoptosis in CAC compared to sCRC, indicating an impaired T-cell function [27]. In contrast, Soh et al. reported lower CD3+ and CD8+ immune infiltration between CAC and sCRC (*n* = 48) within a CAC cohort of comparable size (*n* = 24). However, these groups were not matched regarding tumor stage or localization [14].

In general, a high level of T-cell infiltrates in the mucosa of IBD patients is associated with inflammatory activity [28]. A recent study by Globig et al. found IL-17-producing CD8+ cells enriched in inflamed CD mucosa, contributing to the inflammation [10]. In the tumor-free mucosa of CAC, we found enriched CD8+ infiltrate compared to CRC. We also found that the CD8+ infiltrate in IBD mucosa was significantly enriched compared to tumor tissue and inflamed mucosa.

In the present study, patients with sCRC exhibited significantly elevated FOXP3 levels in early-stage tumors, as confirmed by the data of Soh et al. [14], as well as elevated levels of TIGIT. TIGIT is a coinhibitory molecule similar in function to CTLA4 [29]. In our study, patients in the sCRC group with high levels of FOXP3+ cells had earlier tumor stages than those with low levels. Tregs act suppressively on effector T-cells and can induce an exhausted T-cell phenotype through the expression of inhibitory molecules and the secretion of inhibitory cytokines [30]. Data on the role of Tregs in CAC are sparse and are mainly derived from animal models [31]. Moreover, the role of Tregs in patients with higher numbers of FOXP3+ cells in CAC, similar to sCRC, tends to have a worse prognosis [14]. The inconsistent definition of Tregs and the reliance on FOXP3 as the sole marker for these cells may account for conflicting results in the literature for CRC [32]. Methodological differences, tumor site, stage, and molecular subtype may also contribute to the variability of results, as well as the spatial distance between Treg cells and CD8+ T-cells [33]. 

Furthermore, a low TIGIT count was associated with improved survival in sCRC but not CAC. One study found that TIGIT is particularly abundant on TILs and explicitly identifies the most nonfunctional CD8+ T-cells and FOXP3+ Tregs in mouse tumor tissue [34]. They further stated that the role of TIGIT in suppressing antitumor immune responses is not due to its function in effector CD8+ T-cells but in FOXP3+ Tregs [34]. TIGIT expression on Tregs also suppresses T-cell activation in cancer [29]. TIGIT-expressing Tregs are found at high levels in the microenvironment of various types of cancer, including CRC, and are believed to play a role in suppressing antitumor immune responses [35].

The transcription factor TOX has been identified as a transcriptional and epigenetic regulator for T-cell exhaustion [23]. Recent studies have demonstrated that the mRNA expression of TOX2 and TOX3 is significantly elevated in tumor tissue from individuals with sCRC, suggesting a potential role for these molecules in the development and progression of the disease [36]. We found that CAC exhibited a significantly enriched presence of TIGIT and TOX compared to corresponding tumor-free or even inflamed mucosa. Furthermore, a notable enrichment of TIGIT and TOX counts was observed in the context of sCRC. Notably, sCRC displayed a significantly elevated abundance of TOX+ cells both within the tumor microenvironment and in the adjacent tumor-free mucosa when compared to CAC. Our study first described the expression of the exhaustion markers TOX and TIGIT in the mucosa and tumor of patients with CAC. The results suggest that the expression of TOX does not appear to be enormously increased in CAC (mucosa or tumor) compared to sCRC, in contrast to recent interesting observations of increased numbers of exhausted CD8+ T-cells in the context of IBD [11,13]. T-cell exhaustion might play only a minor role in CAC development. Therefore, further in-depth analyses are needed to understand the differences in exhaustion between tumor-specific T-cells and those in the tumor-free mucosa of CAC patients.

We could confirm the findings of Edin et al., who found an elevated level of CD20+ infiltrate in 316 CRC samples by comparing the right-sided with left-sided tumors in sCRC and CAC [37]. CAC showed significantly improved OS with an increased B-cell count; however, this was not significant for sCRC patients. The univariate analysis also failed to demonstrate B-cell infiltrate as a risk factor for sCRC and CAC. Of note, the univariate Cox regression analysis in this study confirmed the prognostic significance of the lymph node ratio, which is consistent with the findings of Rosenberg et al. [38].

Neutrophils are present in the tumor microenvironment of several types of cancer, and these “tumor-associated neutrophils” (TANs) have many protumor functions, including promoting growth and invasion of the tumor [39]. As an established marker of TANs, CD66b is a protein constitutively expressed by neutrophils and stored within their granules [40,41]. In cancer, a high count of neutrophils is linked to poor overall survival, as a large meta-analysis shows [42]. Higher densities of TANs expressing CD66b were associated with a better outcome in colorectal cancer [43]. However, in our study, no difference was observed for CD66b between CAC and sCRC, and CD66b expression did not impact overall survival in either group. 

In this study, we did not address dendritic cells such as macrophages. Tumor-associated macrophages (TAMs) are a diverse population of immune cells that play a complex role in CRC and probably in CAC prognosis [44]. Macrophages can polarize into M1 and M2 types, with distinct roles. M1 promotes inflammation and has antitumor effects, while M2 has anti-inflammatory properties and supports cancer development [45]. The type and location of TAMs can have different prognostic significance: M2 TAMs have been associated with poor CRC prognosis, and high M1 macrophage and CD68+TAM infiltration is correlated with a better prognostic situation in CRC patients [45]. The transition from UC to CAC involves two key processes, namely inflammation and tumorigenesis, both of which involve macrophages [44]. For example, in the UC inflammatory phase, excessive macrophage infiltration could worsen the inflammation by inducing proinflammatory cytokine production and promoting CAC. Then, before tumor formation, M1 primarily drives inflammation and tumorigenesis. In established CAC, M1 inhibits progression via antitumor immunity, while M2 promotes tumor growth and metastasis [44]. Notably, some studies suggest that M2 not only advances tumors but also raises CAC risk, possibly due to its early-stage tumor-promoting role [46].

Distinct mutational spectra and molecular expression patterns characterize the location (left vs. right) of CRCs [19]. In our study, tumors on the right side exhibited a higher enrichment of FOXP3 and TOX expression when compared to tumors on the left side. This finding suggests a potential correlation between right-sided tumor location and the presence of potentially exhausted T-cells. These results highlight the significance of considering the tumor’s anatomical location when studying the tumor microenvironment. Previous studies focusing on CAC and IBD-associated T-cell exhaustion have not adequately addressed this aspect. Consequently, the absence of consideration for this factor in prior research might elucidate specific disparities observed in our results [10,14].

At present, the clinical significance is, of course, constrained. Our objective was to obtain initial indications of which immune factors might contribute to a notably aggressive course of CAC. Specifically, easily measurable parameters that are detectable through immunohistochemistry could be of great utility in this regard. The next step involves conducting more in-depth investigations, with a focus on lymphocytes, to identify more precise differences between these tumor entities and to improve the role of T-cell exhaustion and tumor prevention in IBD patients. Chronic inflammation promotes the development of colorectal cancer (CRC) and is considered the main cause of CRC associated with inflammatory bowel disease (IBD-CRC) [4]; however, the exact mechanisms involved remain unclear [5]. The primary anti-inflammatory therapy theoretically should reduce the risk of IBD-CRC. Clinical cohort studies have so far demonstrated a chemopreventive effect only for 5-aminosalicylate (5-ASA); however, this effect has not consistently appeared in population-based cohort studies [47]. Especially for the newer available treatments, particularly biologics, their influence on carcinogenesis and, consequently, their effect on the risk of developing CRC during the course of the disease remain uncertain [48].

Our study has some limitations: First, the tissue samples utilized in this study were sourced from a single center and were retrospectively selected for further staining procedures. Therefore, it is essential to consider the potential influence of selection bias. Second, a relatively small number of CAC patients recruited in this study may impose limitations. Hence, survival analyses should be interpreted with caution, especially in this context. Third, the association between the number of immune cells and the use of immunosuppressants or anti-TNF drugs was unclear. Still, we and others [14] could not identify significant differences in the density of immune cells. Fourth, the group of sCRC patients differed from the CAC group in terms of age; it is observed that the onset of CAC occurs at an earlier age than sCRC [3,5]. However, the overall survival rate did not differ significantly in our cohort. Nevertheless, in our recently published meta-analysis, CAC patients showed a worse prognosis regarding OS and cancer-specific survival, probably due to our relatively small sample size [3].

In our study, we took a comprehensive approach by examining multiple cell types by their key markers in tumor-free and inflammatory tissue, along with tumor-infiltrating cells. Despite the rarity of CAC tumors, we were able to conduct a valuable analysis of the tissues, which provided us with the best initial screening option available. The QTiS algorithm was demonstrated to provide a reliable, accurate, and cost-effective quantification of tumor-infiltrating immune stroma cells. This method provides a standardized approach for counting cells [15,17]. This approach allowed us to gather essential insights and laid the foundation for future investigations in this field.

## 5. Conclusions

The study revealed a significant divergence in T-cell exhaustion markers TIGIT and TOX, shedding light on the distinct nature of CAC. Due to the relatively small number of patients, a further investigation is needed to gain deeper insights into the role of immune infiltrates in CAC.

## Figures and Tables

**Table 1:** Clinical characteristics of colitis-associated CRC and sporadic CRC.

	CAC (*n* = 20)	SCRC (*n* = 20)	*p*-Value
Age, years *	50.85 ± 14.48	68.2 ± 11.52	<0.001
Sex			0.45
Female	6 (30%)	3 (15%)	
Male	14 (70%)	17 (85%)	
Sidedness of Tumor			1.00
Left side	8 (40%)	9 (45%)	
Right side	12 (60%)	11 (55%)	
T (AJCC 7th)			0.99
T1	3 (15%)	3 (15%)	
T2	3 (15%)	3 (15%)	
T3	8 (40%)	7 (35%)	
T4	6 (30%)	7 (35%)	
N (AJCC 7th)			0.8
N1	6 (30%)	7 (35%)	
N2	6 (30%)	7 (35%)	
N3	8 (40%)	6 (30%)	
M (AJCC 7th)			0.72
M0	16 (80%)	14 (70%)	
M1	4 (20%)	6 (30%)	
Tumor stage UICC (AJCC 7th)			0.78
I	3 (15%)	4 (20%)	
II	3 (15%)	3 (15%	
III	10 (50%)	7 (35%)	
IV	4 (20%)	6 (30%)	
Grade			0.59
1	1 (5%)	0	
2	8 (40%)	9 (45%)	
3	11 (55%)	11 (55%)	
Neoadjuvant RCT	1 (5%)	2 (10%)	0.54
IBD Type			<0.001
UC	13 (65%)	0	
CD	7 (35%)	0	
None	0	20 (100%)	
CCI *	3.45 ± 2.09	4.50 ± 2.19	0.11
Lymph node ratio *	0.16 ± 0.21	0.15 ± 0.27	0.66
Follow-up months ^#^	30.7 [7–118]	40.1 [4–91]	0.16
OS			

Abbreviations: colitis-associated colorectal cancer (CAC); Charlson Comorbidity Index (CCI); colorectal cancer (CRC); American Joint Committee on Cancer (AJCC); Union of international cancer control (UICC); tumor (T); lymph node (N); Metastasis (M); Inflammatory bowel disease (IBD); Ulcerative colitis (UC); Crohn’s disease (CD), Radiochemotherapy (RCT). Data are represented as *n* (%) unless otherwise annotated. * Age, CCI, and lymph node ratio were presented as mean ± standard deviation. ^#^ Follow-up months was expressed as median (25th percentile–75th percentile).

**Table 2:** A univariate and multivariate Cox regression analysis on eight immunohistochemical markers and other potential risk factors was performed to examine their impact on the overall survival of patients with colitis-associated colorectal cancer (CAC) and sporadic colorectal cancer (sCRC). The *p*-values < 0.10 were defined for the inclusion of covariates in the multivariate Cox regression after the univariate Cox regression. Overall survival refers to the time from the surgical resection to death.

Characteristics	Overall Survival			
	Univariate Cox Analysis		Multivariate Cox Analysis	
	HR (95% CI)	*p*-Value	HR (95% CI)	*p*-Value
CAC (*n* = 20)				
CD3	1.00 (1.00–1.00)	**0.030**	1.00 (0.99–1.00)	0.120
CD4	1.00 (1.00–1.00)	**0.050**	1.00 (1.00–1.00)	0.490
CD8	1.00 (0.99–1.01)	0.630	——	——
Foxp3	0.99 (0.98–1.01)	0.330	——	——
TIGIT	1.00 (0.99–1.00)	0.580	——	——
TOX	1.00 (0.99–1.01)	0.730	——	——
CD20	1.00 (1.00–1.00)	0.150	——	——
CD66b	1.00 (1.00–1.00)	0.490	——	——
Age	1.02 (0.97–1.07)	0.520	——	——
Gender	2.29 (0.47–11.21)	0.310	——	——
Tumor stage	3.3 (0.95–11.43)	**0.060**	1.03 (0.26–4.13)	0.960
Sideness	0.45 (0.12–1.69)	0.240	——	——
Lymph node ratio	39.28 (1.71–902.80)	**0.020**	11.78 (0.14–976.82)	0.270
Charlson Score	1.31 (0.99–1.74)	**0.060**	1.32 (0.89–1.95)	0.170
Sporadic CRC (*n* = 20)				
CD3	1.00 (1.00–1.00)	**0.090**	1.00 (1.00–1.01)	**0.039**
CD4	1.00 (1.00–1.00)	0.426	——	——
CD8	1.00 (1.00–1.01)	0.131	——	——
Foxp3	0.99 (0.98–1.00)	0.188	——	——
TIGIT	1.00 (0.99–1.01)	0.589	——	——
TOX	1.00 (0.99–1.00)	0.710	——	——
CD20	1.00 (1.00–1.00)	0.908	——	——
CD66b	1.00 (1.00–1.00)	0.926	——	——
Age	0.99 (0.94–1.05)	0.803	——	——
Gender	0.38 (0.07–2.07)	0.262	——	——
Tumor stage	2.54 (0.93–6.91)	**0.069**	2.94 (1.18–7.33)	**0.021**
Sidedness	0.68 (0.14–3.24)	0.633	——	——
Lymph node ratio	31.00 (0.28–3486.38)	0.154	——	——
Charlson Score	1.09 (0.73–1.62)	0.688	——	——

*p*-values less than 0.100 were marked with the bold in the Cox regression analysis.

## Data Availability

Data and analytical methods will be made available upon reasonable request and in agreement with patient data protection legislation.

## Supplementary Materials

The following supporting information can be downloaded at: https://www.mdpi.com/article/10.3390/cancers15194743/s1, Figure S1: (**A**) To compare the overall survival between sCRC and CAC patients, the Log-rank test was employed. (**B**) A correlation analysis was conducted using the Pearson correlation to assess the relationship between immune infiltrations and clinical test parameters in both CAC and sCRC patients; Figure S2: Comparing the immune cell composition between ulcerative colitis associated (UC)-CRC, Crohn’s disease associated (CD)-CRC and sporadic (s)CRC in the tumor tissue; Figure S3: displays Receiver Operating Characteristic (ROC) plots, which are used to assess the predictive capability of various immune infiltrations for overall survival (OS) in CAC patients; Figure S4: A prognostic analysis of immune infiltrations in sporadic Colorectal Cancer (sCRC) patients after using the Survminer R package to select the best cut-off point for classifying the patient cohort into high or low-infiltration groups; Figure S5: displays Receiver Operating Characteristic (ROC) plots, which are used to assess the predictive capability of various immune infiltrations for overall survival (OS) in sCRC patients; Table S1. The detailed information of the primary antibodies applied in immunohistochemistry staining; Table S2: Demonstrates the counting threshold values for classifying a population as having a “high” level of marker expression as defined using the Survminer R package to select the best cut-off counts (in Figure 5 and Figure S4).
