# Supplementary material for: Differential Immune Infiltration Profiles in Colitis-Associated Colorectal Cancer versus Sporadic Colorectal Cancer
Source: Cancers (Basel). 2023 Sep 27;15(19):4743. doi: 10.3390/cancers15194743 (PMC10571767; doi:10.3390/cancers15194743)
Supplement: Supplementary file 1 [file cancers-15-04743-s001.zip › cancers-2583370-SI.pdf]

Supplementary Material

Supplementary Figures

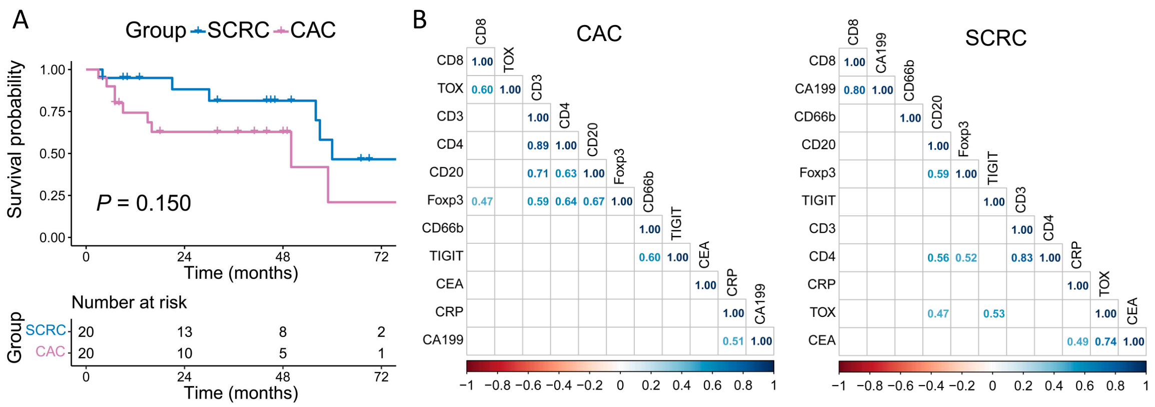

Supplementary Figure S1 (A) To compare the overall survival between sCRC and CAC patients, the Log-rank test was employed. (B) A correlation analysis was conducted using the Pearson correlation to assess the relationship between immune infiltrations and clinical test parameters in both CAC and sCRC patients. The resulting correlation coefficients (r) were depicted, while blank entries represent cases where the associated p-value exceeded 0.05, indicating a lack of significant correlation. Abbreviations: CAC, colitis-associated colorectal cancer; SCRC, sporadic colorectal cancer;

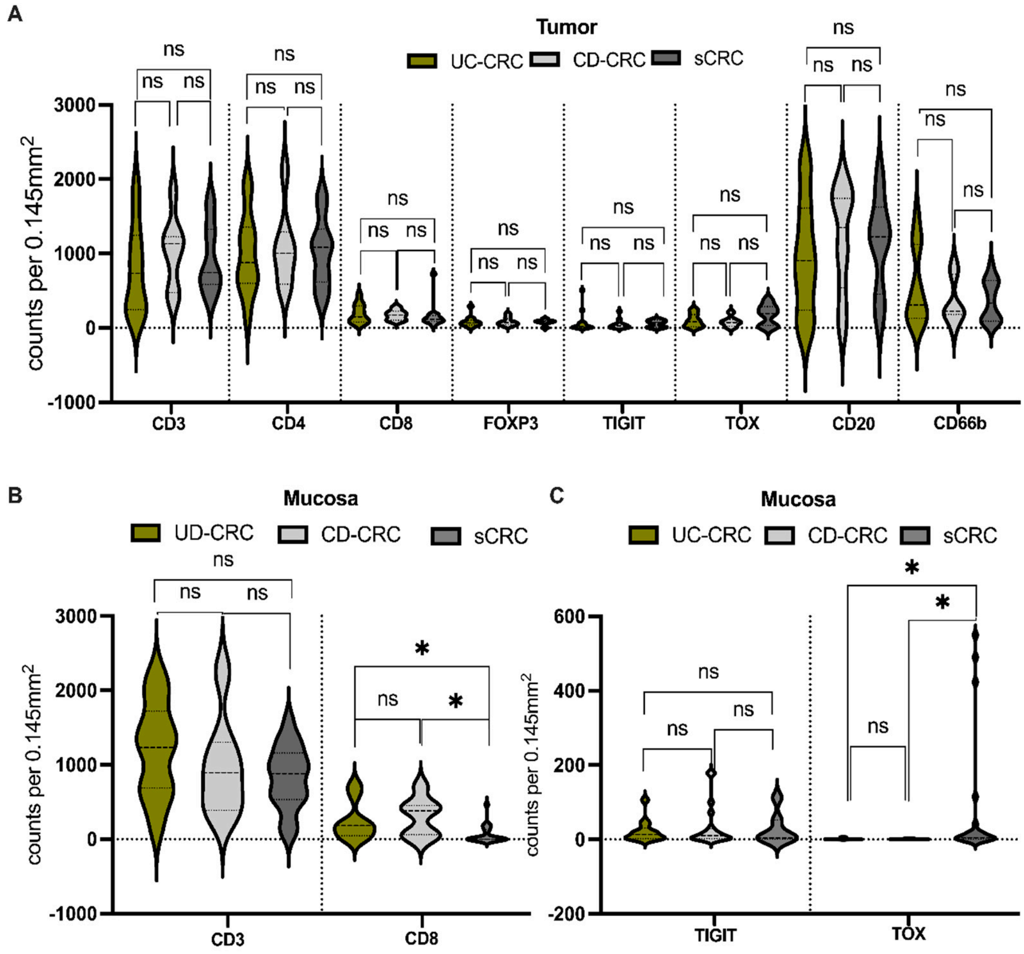

Supplementary Figure S2 Comparing the immune cell composition between ulcerative colitis associated (UC)-CRC, Crohn's disease associated (CD)-CRC and sporadic (s)CRC in the tumor tissue (A) with violin plots demonstrating the tumor infiltration levels of 8 immune markers in UC-CRC (n = 13), CD-CRC (n = 7), and sCRC (n = 20). In tumor-free mucosa, CD3+ and CD8+ infiltrates (B) were compared among the above groups, TIGIT+ and TOX+ cell-counts (C) in the tumor-free mucosa were compared among the above groups.

Multiple comparisons test was used to correct the P value of the comparison of the markers between UC-CRC, CD-CRC and sCRC (ns; P ≥ 0.05; P < 0.05, ** P < 0.01, *** P < 0.001, ns=not significant).

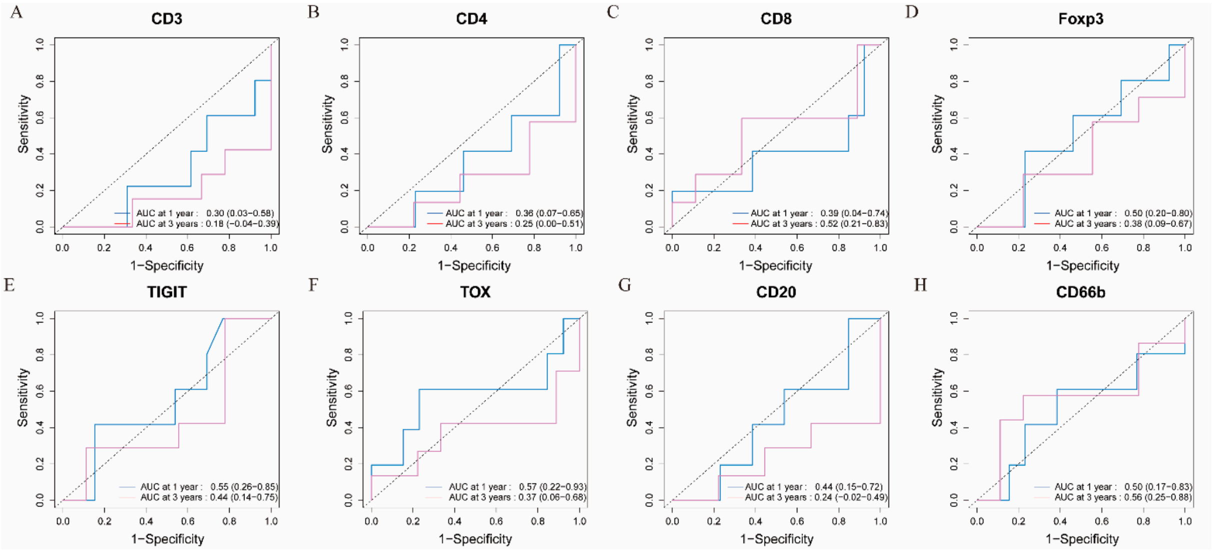

Supplementary Figure S3 displays Receiver Operating Characteristic (ROC) plots, which are used to assess the predictive capability of various immune infiltrations for overall survival (OS) in CAC patients. The ROC plots provide a visual representation of the performance of these immune infiltrations as prognostic factors for OS in CAC.

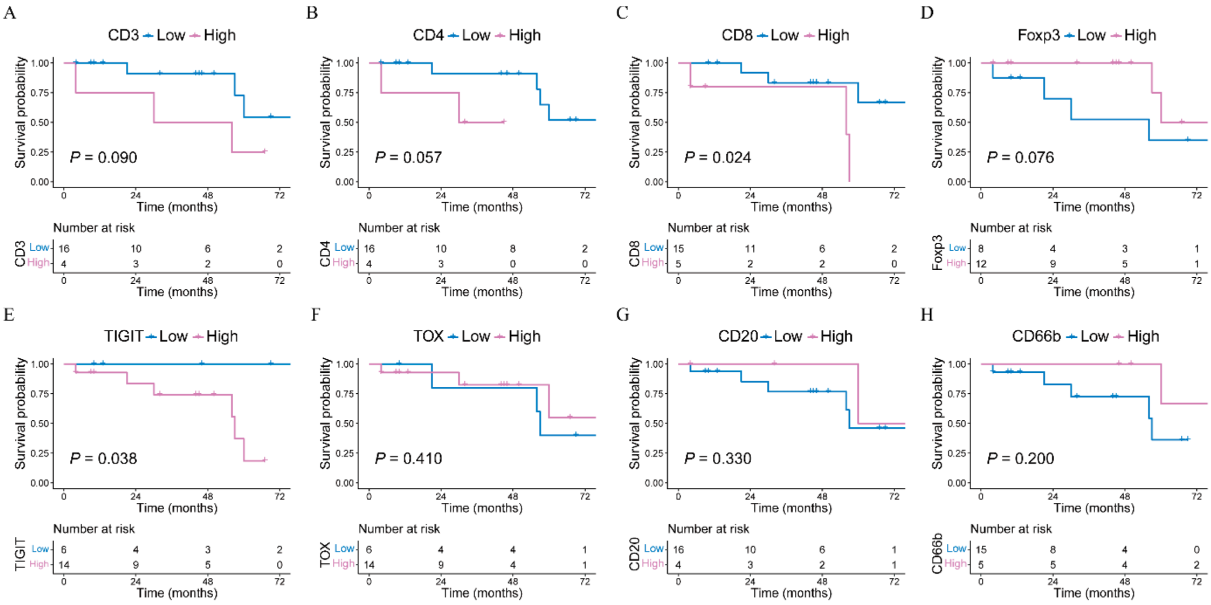

Supplementary Figure S4 A prognostic analysis of immune infiltrations in sporadic Colorectal Cancer (sCRC) patients after using the Survminer R package to select the best cut-off point for classifying the patient cohort into high or low-infiltration groups.

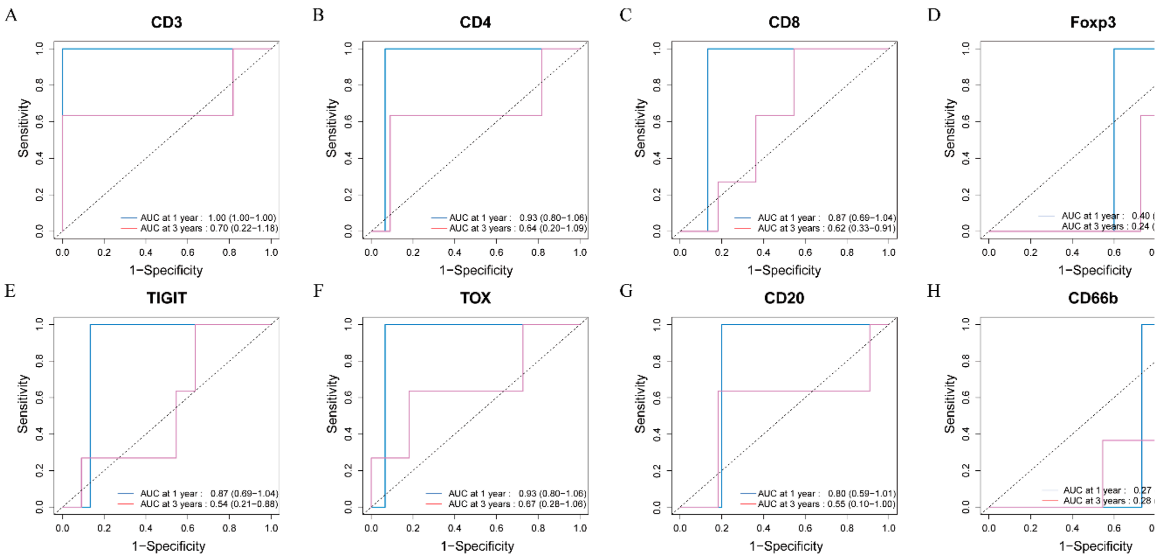

Supplementary Figure S5 displays Receiver Operating Characteristic (ROC) plots, which are used to assess the predictive capability of various immune infiltrations for overall survival (OS) in sCRC patients. The ROC plots provide a visual representation of the performance of these immune infiltrations as prognostic factors for OS in sCRC.

Supplementary Tables

Antibodies	Dilution	Catalogue number	Company	City	Country
Markers					
CD3	1:100	Ab5690	Abcam	Cambridge	UK
CD4	1:600	Ab133616	Abcam	Cambridge	UK
CD8	1:100	Ab17147	Abcam	Cambridge	UK
Foxp3	1:400	Ab20034	Abcam	Cambridge	UK
TIGIT	1:600	Ab243903	Abcam	Cambridge	UK
TOX	1:200	HPA018322	Sigma-Aldrich	Burlington, Massachusetts	USA
CD20	1:400	Ab78237	Abcam	Cambridge	UK
CD66b	1:400	305102	Biolegend	San Diego, California	USA
Isotype Control					
IgG	—	Ab37415	Abcam	Cambridge	UK
IgG1, κ	—	Ab91353	Abcam	Cambridge	UK
IgM, κ	—	401601	Biolegend	San Diego, California	USA

Supplementary Table S1. The detailed information of the primary antibodies applied in immunohistochemistry staining.

Abbreviations: FOXP3, Forkhead box P3; TIGIT, T cell immunoreceptor with immunoglobulin and ITIM domain; TOX, thymocyte selection-associated HMG BOX; UK, United Kingdom; USA, United States of America.

Variables	CAC	sCRC
CD3	273.00	1280.67
CD4	1058.00	1338.33
CD8	108.67	170.00
Foxp3	84.00	73.67
TIGIT	39.67	8.00
TOX	115.00	60.33
CD20	149.33	1751.33
CD66b	723.67	591.00

Supplementary Table S2: Demonstrates the counting threshold values for classifying a population as having a "high" level of marker expression as defined using the Survminer R package to select the best cut-off counts (in Figure 5 and Suppl. Figure S4).
